# Advances in Understanding the Human Gut Microbiota and Its Implication in Pediatric Celiac Disease—A Narrative Review

**DOI:** 10.3390/nu15112499

**Published:** 2023-05-27

**Authors:** Vasile Valeriu Lupu, Laura Mihaela Trandafir, Anca Adam Raileanu, Cristina Maria Mihai, Ionela Daniela Morariu, Iuliana Magdalena Starcea, Adriana Mocanu, Lacramioara Ionela Butnariu, Gabriela Stoleriu, Delia Lidia Salaru, Tatiana Chisnoiu, Dragos Munteanu, Costica Mitrofan, Ancuta Lupu

**Affiliations:** 1 Department of Pediatrics, “Grigore T. Popa” University of Medicine and Pharmacy, 700115 Iasi, Romania; 2 Pediatrics, Ovidius University, 900470 Constanta, Romania; 3 Faculty of Pharmacy, “Grigore T. Popa” University of Medicine and Pharmacy, 700115 Iasi, Romania; 4 Genetics, “Grigore T. Popa” University of Medicine and Pharmacy, 700115 Iasi, Romania; 5 Clinical Medical Department, Faculty of Medicine and Pharmacy, “Dunarea de Jos” University of Galati, 800008 Galati, Romania; 6 Faculty of General Medicine, “Grigore T. Popa” University of Medicine and Pharmacy, 700115 Iasi, Romania

**Keywords:** celiac disease, pediatric, gut microbiota, dysbiosis, microbiota modulation

## Abstract

Celiac disease (CD) is a multifactorial disorder, defined by a complex interplay of genetic and environmental factors. Both genetic predisposition and dietary exposure to gluten are essential factors in triggering CD. However, there is proof that their presence is necessary, but not sufficient, for disease development. Through gut microbiota modulation, several additional environmental factors have shown their potential role as co-factors in CD pathogenesis. The aim of this review is to illustrate the possible mechanisms that stand behind the gut microbiota’s involvement in CD pathogenesis. Furthermore, we discuss microbiota manipulation’s potential role as both a preventative and therapeutic option. The available literature provides evidence that even before CD onset, factors including cesarean birth and formula feeding, as well as intestinal infection exposure, amplify the risk of CD in genetically predisposed individuals, due to their influence on the intestinal microbiome composition. Active CD was associated with elevated levels of several Gram-negative bacterial genera, including *Bacteroides*, *Escherichia*, and *Prevotella*, while beneficial bacteria such as lactobacilli and bifidobacteria were less abundant. Viral and fungal dysbiosis has also been described in CD, evidencing specific taxa alteration. A gluten-free diet (GFD) may improve the clinical symptoms and duodenal histopathology, but the persistence of intestinal dysbiosis in CD children under a GFD urges the need for additional therapy. Probiotics, prebiotics, and fecal microbial transplant have demonstrated their efficacy in restoring gut microbiota eubiosis in adult CD patients; however, their efficacy and safety as adjunctive therapies to a GFD in pediatric patients needs further investigation.

## 1. Introduction

Celiac disease (CD) is an autoimmune-mediated, chronic systemic disease, triggered by alimentary exposure to gluten, in genetically predisposed individuals [1]. Although, in the past, CD was considered a rare disease, recent epidemiologic evidence offers proof of an increase in CD’s incidence, both in adults and children, affecting 0.7% of the general population and almost 1% of the pediatric population [2,3]. The incidence of CD is twice as high in children compared to adults, with women being 1.5 times more likely to be affected than men [4]. 

CD has been mentioned numerous times across history, but it was only at the end of the 1940s when it was defined as an independent entity. The pediatrician Willem-Karel Dicke first characterized CD, together with the effects of the consumption of bread containing gluten [5]. The histologic anomalies associated with CD were described by Paulley in 1954, after analyzing surgical samples that were taken from patients who were known to have CD [6]. In 1964, Berger and colleagues [7] first reported the presence of anti-gliadin antibodies (AGAs), while Falchuk, in 1972, described the association of CD and a special HLA haplotype [8]. The first guidelines referring to CD diagnosis were published in 1969 and updated periodically by the European Society for Paediatric Gastroenterology and Nutrition (ESPGHAN) [9,10,11,12]. Currently, diagnosis is based on serum IgA anti-transglutaminase (tTG) auto-antibody detection, in association with duodenal villous atrophy, while treatment relies on an accurate gluten-free diet (GFD) [9]. Despite extensive research, there are still gaps in knowledge regarding CD pathogenesis, as well as in its therapeutic options. 

The gastrointestinal tract hosts the largest number of microorganisms in the human body, including bacteria, fungi, viruses, archaea, and protozoa, and its composition changes continuously. Microbiome development begins during prenatal life and proceeds throughout senescence, depending on each individual’s exposure to several factors [13,14,15]. Gut microbiota is known to be responsible for many physiological processes of the human body, including metabolism, nutrition, and immune system function [13]. During the last decade, our comprehension of the microbiome and its complex synergy with host cells and organisms has increased exponentially, due to the wide availability of modern technology, including next-generation sequencing. There is emerging evidence that gut-associated microorganisms also contribute to CD pathogenesis and disease progression. This review summarizes the current CD-related microbiome data in order to obtain a better understanding of the possible mechanisms that stand behind the gut microbiota’s involvement in CD pathogenesis. Furthermore, as a component of personalized medical management, we discuss microbiota manipulation’s potential role as both a preventative and therapeutic option.

## 2. Genetic and Environmental Determinants of Celiac Disease

Genetics represent an important factor in CD, as genetic susceptibility associated to a gluten-rich diet leads to different geographical variations of CD prevalence. HLA testing in CD has a low positive predictive value, while its negative value has a high predictive validity [16]. HLA-DQ2/8, which is characteristic to CD, can also be found in 40% of the general population, suggesting that genetics are not enough for CD progression [17]. However, 90% of CD patients have an HLA-DQ 2.5 genotype, while the other 10% share an HLA-DQ 2.2 or HLA-DQ 8 version [18]. These genotypes and their different associations can be correlated to distinct clinical features and might play a role in predicting disease severity [18]. 

Gluten, as already stated, represents another condition that is necessary for CD induction. As a protein mixture, gluten’s main components are gliadin and glutein. Gliadin is the principal antigen that triggers CD thorough its components glutamine and proline. When they arrive in the small intestine, these proline-rich peptides have a longer period of degradation, increasing the chance of activating an immune response. Once both the innate and the adaptive responses are triggered, this will further lead to crypt hyperplasia, villus atrophy, and to the infiltration of the intestinal epithelial inflammatory cells [19]. In addition, these pathological modifications will be followed by an increase in intestinal permeability and to intestinal epithelial cell destruction, clinically translated as diarrhea, emaciation, abdominal pain, abdominal meteorism, and dermatitis herpetiformis, as well as features of CD pathogenesis [16].

**Figure 1:**
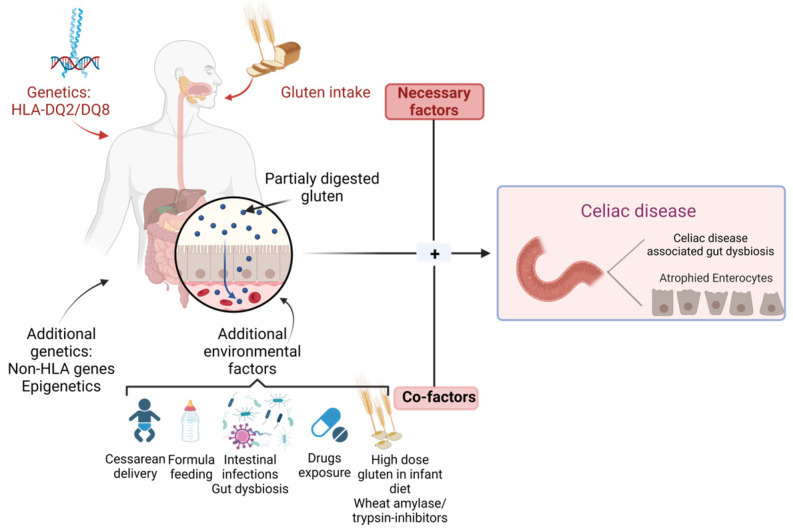
Genetic and environmental factors in CD.

However, the current medical literature sustains the fact that a gluten-rich diet in a genetically predisposed individual does not offer full premises for CD autoimmunity (CDA) development, indicating the role of additional environmental factors, as well as gluten, in the disease pathological process [20]. CD pathogenesis seems to involve several factors, including the type of birth, infant feeding techniques, and intestinal infections, as well as drug exposure, due to their ability to influence microbiota composition [1], as shown in Figure 1.

It is well known that the human microbiome has a great influence on one’s state of health, as well as their state of disease. Gut bacterial community dysbiosis can alter the gastrointestinal microecological environment, turning into a pathogenic factor element for a broad spectrum of disorders, such as gastrointestinal, cardiac, respiratory, neurological, and metabolic diseases [13,21,22]. The recent advances in human microbiome study have offered evidence that diet is a major determinant of the intestinal microbiota’s composition and function, and gluten can have an important influence on the gut microbiota’s stability.

## 3. Gut Microbiota Profile in CD Progressors

By mediating the interactions between the immune system of the host and gluten/environmental factors, the gut microbiome seems to play a key role in CD pathogenesis [23].

Several studies have explored the relationship between the alteration of the intestinal microbiota and different environmental factors in subjects at risk of developing CD. Evaluating the gut microbiota composition of children at risk of developing CD before the onset of the disease might be useful in identifying possible CD progression markers. Pozo-Rubio and colleagues [24] analyzed the fecal microbial composition of children at risk of CD and reported some association between some pre-selected microbial taxa and the delivery mode, feeding practices of the infant, the administration of the rotavirus vaccine, and antibiotic exposure [24]. 

Similarly, Leonard et al. [25] focused their efforts on analyzing the influence of genetic and environmental risk factors on the composition of the intestinal microbiota, before solid food and gluten introduction, in infants known to be at risk for CD. In their report, infants who were genetically predisposed to CD displayed a decreased amount of several *Coprococcus*, *Streptococcus*, *Parabacteroides*, and *Veillonella* species and *Clostridium perfringens* at four and six months of age [25], which was similar to the results of Hov and colleagues [26], who also identified lower levels of *Coprocococcus* in individuals genetically at risk of different autoimmune disorders [26]. Infants with a genetic risk of CD presented increased amounts of *Bacteroides* and *Enterococcus* species, as proven in several different studies [25,27,28]. As for the type of delivery, the intestinal microbiota of children delivered via cesarean section was characterized by an elevated abundance of *Enterococcus faecalis* at 3 months after birth, while several species of *Parabacteroides* and *Bacteroides* were reported in lower amounts compared to infants born through vaginal delivery, at all time points [25]. The association of cesarean section with decreased levels of *Bacteroides vulgatus* and *Bacteroides dorei* strengthens the influence of the birth method on CD risk. These beneficial species, in increased amounts, are reported to lower the production of microbial lipopolysaccharide, leading to an improvement in the host’s immune response [29]. Furthermore, infants born via cesarean section displayed decreased folate synthesis and riboflavin metabolism at the ages of four and six months, changes that might be associated with an altered immune response to viral aggression and a reduced natural killer cell activity [25,29]. Some authors correlate the last finding with the increased probability of developing type 1 diabetes, a disorder that is often associated with CD [30]. 

Consistent with several studies available in the literature, exclusive formula feeding is associated with elevated levels of *Lachnospiraceae bacterium* and *Rumminococcus gnavus*, previously associated with colonic inflammation, diabetes, and allergy disorders [25,31,32,33]. Antibiotic exposure was found to be related to an elevated level of *Bacteroides thetaiotaomicron* in the intestinal microbiota of four- to six-month-old infants at risk of CD, which is a bacterium known to be an important metabolizer of polysaccharides [25,34].

In a recent cross-sectional and longitudinal metagenomic analysis of the fecal microbial communities of infants at risk of CD, Leonard and colleagues [23] evaluated the gut microbiota composition and its associated metabolites in infants who progressed to CD, in comparison to matching non-affected controls. The surveillance started 18 months before the disease onset. The cross-sectional analysis that was performed at the moment of the onset reported differences in the abundance of several bacterial strains and derived metabolites in the infants who developed CD compared to the controls, while the pathway abundance and microbial species evaluation revealed no modification. *Bacteroides vulgatus* and *B. uniformis* were found in decreased levels, which was further associated with a reduced efficacy of the immune defense mechanisms. The longitudinal study before the disease onset, however, described elevated levels of different microbial species, strains, and metabolites. Some microbial species were also reported to be associated with some other autoimmune disorders, suggesting that they could be used as possible biomarkers in autoimmune disease identification. *Dialister invisus* was found in elevated levels at all of the time points when compared to its levels at CD onset [23], while other studies reported an increased abundance in children with pre-type-1 diabetes and individuals who further developed CD [35,36].

The number of *Parabacteroides* species was also higher before CD onset, similar to other autoimmune disorders, such as type 1 diabetes and Behcet’s disease [37,38]. *Lachnospiraceae bacterium* is another representative whose abundance was found to be higher prior to CD onset. *L. bacterium* colonization in mice was linked to diabetes and obesity in genetically predisposed subjects, also being capable of inducing colonic inflammation [39,40]. In contrast, bacteria with anti-inflammatory proprieties, including *Streptococcus thermopilus* and *Faecalibacterium prausnitzii*, were found to be decreased in abundance in patients before CD onset [23].

Immunoglobulin A (IgA) is the main representative antibody of mucosal immunity. Recent evidence has revealed that, at the age of five, the humoral immune response of CD progressors appeared altered, as such individuals displayed higher levels of IgA positive-coated bacteria and unique targets of IgA in their intestinal microbiota, in comparison with controls [35]. An important observation is that the top targets of the immune mucosal response in these patients are reported to be intestinal-barrier-protective commensals [41]. 

Another interesting finding in CD progressors is that they have an increased level of taurodeoycholic acid (TDCA) in their plasma [35]. TDCA is a microbiota-derived metabolite that has been shown to stimulate inflammation and cause villous atrophy in the small intestines of mice [42]. Its plasma levels could be used as an early marker for CD diagnosis, while TDCA-producing bacteria could serve as a target in a microbiota-modulation strategy of treatment [35]. The main findings regarding the microbiota profile of children at risk of CD, before disease onset, are summarized in Table 1.

## 4. Gut Microbiota Profile at CD Onset

The fecal microbiota of children with CD seems to be defined by a decrease in the abundance of enterococci, lactobacilli, and bifidobacteria [45]. In their study, El Mouzan and colleagues [46] reported that CD patients display lower levels of the phylum Actinomycetota, mainly representatives of the genus *Bifidobacterium*, which are bacteria with immunomodulatory effects, often used as probiotics. *Roseburia* and *Lachnospiraceae* species, also known as beneficial bacteria, were found in decreased abundance in newly diagnosed CD children. As for the species that were found in an increased in abundance in these children, elevated levels of *Subdoligranulum* species were noted on several occasions, including before disease onset in children with an increased genetic risk of CD [25,46,47]. 

Furthermore, intestinal microbial diversity might vary in CD patients according to their clinical features [48]. The microbiota of patients known to have CD associated with gastrointestinal symptomatology is mainly represented by the Pseudomonadota phylum and decreased microbial diversity, whereas the Bacillota phylum dominates the microbiota of patients with dyspepsia or dermatitis herpetiformis as their main manifestation [49]. These findings offer evidence that intestinal dysbiosis can have an essential role in CD pathogenesis and clinical course. 

Di Biase and colleagues [50], in their pilot study, tried to identify a pattern between the gut microbiota composition and the associated clinical elements present in children at CD onset. After analyzing the stool and duodenal mucosa samples of CD patients in comparison with healthy controls, it was reported that the duodenal microbiota of patients with CD is mainly represented by the *Enterobacteriaceae* family, followed by the Bacteroidota phylum and *Streptococcus* species as major representatives. The stool samples of these patients, on the other hand, are characterized by decreased levels of genus such as *Akkermansia*, *Bacteroides*, and *Prevotella*, and a reduction in levels of the *Staphylococcaceae* family [50]. Furthermore, the authors have pointed out a possible correlation between the presence of abdominal pain in some CD patients and elevated levels of pro-inflammatory microbiota such as *Enterobacteriaceae* and *Bacillaceae* family representatives. In addition to these findings, the levels of bacterial-derived metabolites such as short-chain fatty acids (SCFAs) and *Bacteroides fragilis*-derived polysaccharide A also appeared to be modified in CD patients [50].

The recent data suggest that gut-microbiota-associated dysbiosis could be used in the diagnosis process of CD. A group of researchers that evaluated the predictive power of fecal microbial dysbiosis in CD diagnosis reported that a combination of viruses and bacteria indicated an important predictive power in CD diagnosis, whereas, in mucosal samples, the strongest predictive power was obtained when the bacterial communities were analyzed alone. Compared to healthy controls’ microbiota composition, CD patients could be identified by the reduction in *Burkholderiales bacterium 1-1-47* and *Bacteroides intestinalis* levels in their fecal samples, or by *Human endogenous retrovirus K* in their duodenal mucosa specimens [51]. The reduced abundance of the two bacteria, with roles in degrading dietary fibers and reducing the gluten content of aliments, could be used to generate new therapeutic strategies [46]. The role of *Human endogenous retrovirus K* in CD patients, however, needs further investigation, as this group of viruses can present both damaging and protective actions, depending on their levels [52].

It is well known that fungi are able to relate with the human immune system and with bacteria. The current evidence sustains their role in gastrointestinal disorders, including irritable bowel syndrome and inflammatory bowel disease [53,54]. A study of fungal dysbiosis recently reported in children suggests that fungi might also be an important element in CD pathogenesis. In a metagenomic analysis of mucosal and fecal samples of children known to have CD, taxa such as *Saccharomyces cerevisiae* and *Saccharomycetaceae* were reported to be more abundant in the fecal samples, whereas *Pichiaceae* and *Pichia kudriavzevii* were found in decreased abundance. The mucosal samples were more abundant in taxa including *Sacchharomycetes* and *Candida*, while *Pneumocystis* and *Pneumocystis jirovecii* levels were decreased compared to controls [55]. 

*Saccharomyces cerevisiae* might represent an important factor in CD-associated dysbiosis. Found in increased levels in patients with CD, several studies have noted its reduction or complete disappearance in patients under a gluten-free diet, suggesting its potential role in CD pathogenesis [56,57]. 

*Candida albicans* is one of the yeasts whose immunologic involvement in CD has been the subject of research. It seems that *C. albicans* hyphal wall protein 1 can be covalently associated with endomysium components and to tissue transglutaminase, due to its amino acid sequences, which are similar to CD-related gamma-gliadin and alpha-gliadin T-cell epitopes [58]. Several studies have confirmed the correlation between CD and *C. albicans*, indicating a possible connection with CD initiation and progression [55,59,60].

Although less studied, the human virome represents an important element of the gut microbiome, with a possible implication in the pathogenesis of several digestive immune-mediated disorders. In a study by El Mouzan and colleagues [61], both fecal and duodenal mucosa samples that were obtained from children with CD were characterized by the presence of a viral dysbiosis when compared to non-celiac controls. While the analysis of the mucosal samples found no important association with CD and any viral species, the stool samples were reported to be abundant in species such as *Enterobacteria phage mEpX1*, *Human polyomavirus 2*, and *Human polyomavirus 2*, whereas *Streptococcus phage Abc2* and *Lactococcus phages ul36* were less abundant [61]. Although, so far, no causality has been found between CD and the intestinal virome, the strong association among them raises the suspicion of an important role of viruses, particularly bacteriophages, in CD pathogenesis. The main findings regarding the microbiota profile of children at CD onset are summarized in Table 2.

## 5. CD Prevention Strategies

In order to prevent CD, several gluten interventions have been recommended during recent years. Breastfeeding was thought to have a protective effect against CD, and an early introduction of gluten during a breastfeeding period in infants from four to six months of age was expected to have beneficial outcomes on disease onset [65].

As for dietary intervention in children with a known genetic risk of CD, several clinical trials have evaluated the outcomes of a delayed gluten introduction at 6 and 12 months of age. The BABYDIET clinical trial observed its subjects for 3 and 8 years, concluding that, in genetically at-risk children, a delayed gluten introduction at the age of 12 months does not offer a reduction in the risk of celiac disease autoimmunity (CDA) or an impact on the persistence of tTGA positivity [66,67]. However, there is evidence that a delayed exposure to gluten might lead to a delayed onset of the disease [68,69], while an early introduction of a small, fixed amount of gluten in infants with a known genetic risk reported no effect on disease onset [70].

Data from children with disease onset by the age of six years have revealed that these children had a rich gluten-containing diet after two years of age [71], whereas high gluten consumption during the second year of life increased the risk of CD [72,73]. Two ongoing clinical trials are evaluating the outcome of a late introduction of gluten/a restricted gluten intake up to an age of three and five years old, respectivley. The researchers behind these studies aim to obtain CD prevention, or at least to delay the disease onset to an older age, in children at genetic risk [74]. Furthermore, the Prevention Celiaki i Skåne (PreCiSe) study evaluated whether the intake of a daily probiotic during the first 3 years of life would prevent CD onset up to the age of seven, in comparison with keeping a GFD, or with no dietary intervention. Two strains of Lactobacillus (*Lactobacillus plantarum* HEAL9 and *Lactobacillus paracasei* 8700:2) were chosen, due to their positive actions on the intestinal environment, to modify the underlying mechanisms of CD pathogenesis. The use of *Lactobacillus plantarum* HEAL9 aims to restore the normal permeability of the intestinal mucosa, while *Lactobacillus paracasei* 8700:2 exerts its immunomodulatory effects by regulatory T cells stimulation [75,76].

On several occasions, it has been suggested that viral infectious episodes during the period of inducing tolerance to gluten could have a significant impact on the risk of developing CD [74]. Although the pathophysiologic mechanism that stands behind the viral immune activation of T cells against gluten peptides and the activation of B cells to produce tTGA has not currently been brought to light, there is proof of a strong correlation between viral exposure and celiac disease autoimmunity (CDA) development. An increased exposure to enterovirus B during a child’s first 2 years of life might elevate the risk of CDA, while a diet rich in gluten, in association with an enterovirus B infection history, would offer a cumulative effect regarding the risk of CD [77]. While children who have experienced rotavirus infections have an increased risk of CDA, those who received a rotavirus vaccination seem to be protected from CDA, as long as gluten is introduced in their alimentation before their 6th month of life, suggesting that limiting an infant’s viral exposure might have a beneficial influence on CD risk [78,79].

## 6. Modulation of Gut Microbiota as a Potential Target in Celiac Disease

Dysbiosis was proved to be an important factor in CD pathogenesis and progression. Dietary exclusion of gluten, the key therapeutic option for CD, is not able to fully restore the disrupted intestinal microbiota of CD patients, even after long periods of adherence to a gluten-free diet [45,63]. The available evidence of gut dysbiosis in patients under a GFD is summarized in Table 3.

**Figure 2:**
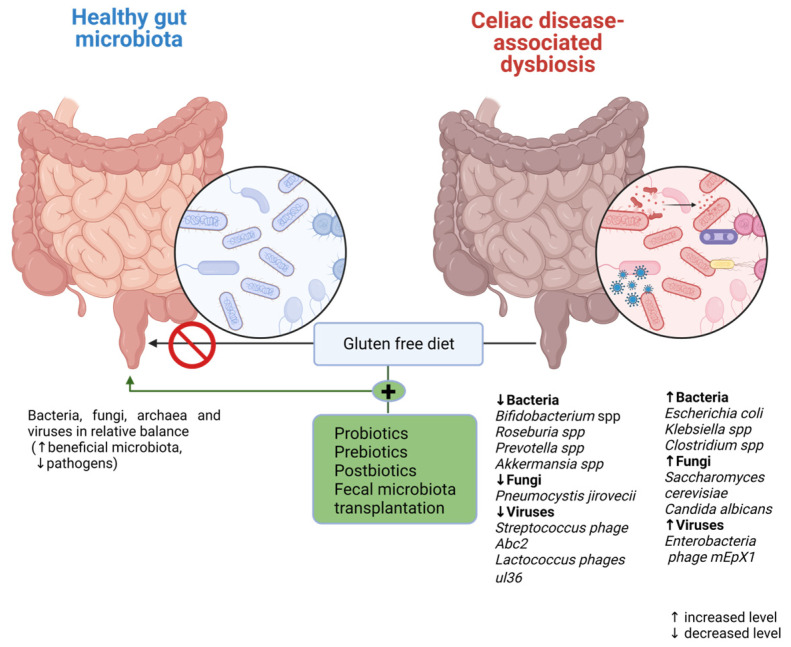
Microbiota modulation therapeutic options in CD.

Currently, there are many available methods of modulating the CD-associated dysbiotic intestinal microbiota in adult patients, including dietary interventions (prebiotics, probiotics, and postbiotics are also included) and fecal transplantation, as shown in Figure 2. However, in pediatric patients, several reports from the available literature have place diet modification (GFD) and probiotic and probiotic use as the main options for the modulation of the gut microbiota.

*Bifidobacterium* strains have shown their potential as probiotics in restoring the normal *Bacillota/Bacteroidota* ratio in children with CD by increasing *Bacillota* phylum abundance. Furthermore, *Bifidobacterium breve* BR03 and B632 administration in CD patients under a GFD restored *Lactobacillaceae* family levels close to those reported in healthy individuals [80]. The re–establishment of the *Bacillota/Bacteroidota* ratio was negatively associated with TNF-alpha serum levels after probiotic intake [81,82,83].

*Bifidobacterium longum* CECT 7347 supplementation in children with new-onset CD following a GFD was evaluated in a double-blind, randomized, placebo-controlled 3-month-intervention trial. The probiotic administration resulted in positive effects on the children’s growth; furthermore, there was no significant difference in gastrointestinal symptoms in the treated CD versus the placebo. Moreover, in the treated patients, there was a decrease in *B. fragilis* fecal and sIgA levels in the fecal samples. As for the TNF-alpha serum levels, only a slight reduction was obtained after bifidobacteria administration [82].

*Bifidobacterium breve* BR03 (DSM 16604) and *Bifidobacterium breve* B632 (DSM 24706) administration in CD children under a GFD resulted in a decreased serum level of the pro-inflammatory cytokine TNF-alpha [81,83]. Although the beneficial effect lasts only during the probiotic administration period [81], lowering TNF-alpha is important in CD patients, as these probiotic strains have the capacity to decrease both intestinal and systemic complications of the disease [82]. 

Prebiotic use has shown its potential in restoring intestinal homeostasis in CD patients. A randomized placebo-controlled trial evaluated the effect of oligofructose-enriched inulin (Synergy 1) supplementation in CD children under a GFD compared to a placebo. After 3 months, the patients receiving the prebiotic presented a significant increase in *Bifidobacterium* count. Furthermore, prebiotic administration increased acetate and butyrate fecal levels, evidence of the stimulation of bacterial metabolite production in CD patients [84]. 

These promising results are encouraging further research, as there is a lack of clinical trials in children with CD. There is a need to generate new evidence regarding the efficacy and safety of these methods of microbiota modulation, since a GFD alone is not enough to re-establish gut microbiota eubiosis.

## 7. Limitations in the Study of Gut Microbiota and CD

Progress has been made in understanding the complex relationship between gut microbiota and CD, due to the advances in the study of the microbiome’s structure and function. However, significant gaps still characterize our knowledge regarding the implication of the gut microbiota in pediatric celiac disease pathogenesis. Most frequently, this is due to the reduced amount of existing prospective and cross-sectional studies, their small number of participants, and their different applied methodologies (16s ribosomal RNA sequencing, fluorescence in situ hybridization-PCR assay, and denaturing gradient gel electrophoresis). This lack of uniformity leads to different results, which interferes with the aim of identifying a distinctive microbial signature in children with CD. Recognizing early changes in the composition of the intestinal microbiota of children who are genetically susceptible to CD and monitoring the influence of several environmental factors on the gut microbiota’s profile over time is another challenge in CD study that would benefit from further research, as these results could be used to develop a future prevention strategy. With a gluten-free diet being the only available treatment for CD, there is a need for additional therapies that would improve patients’ symptoms and quality of life. In adult services, several therapeutic approaches based on microbiota modulation have already proved their efficacy and safety, and their introduction in pediatric practice represents a future challenge for scientists interested in CD research.

## 8. Conclusions

Recent advances in human microbiome research offer evidence of gut-associated microbiota involvement in CD pathogenesis. Children with CD display an abundance of several Gram-negative bacterial genera, including *Bacteroides*, *Escherichia*, and *Prevotella*, a reduction in beneficial bacteria such as lactobacilli and bifidobacteria, and associated fungal and viral dysbiosis. Furthermore, changes in the microbiota’s normal composition can be identified even before disease onset in children who are at risk of CD development. This comes as evidence of how several environmental factors influence the gut microbiota, increasing the risk of CD. Despite being the only way to improve clinical symptoms and duodenal histopathology, a GFD is not able to fully recover the normal composition of the gut microbiota. The findings associated with gut microbiota research in CD patients have led to the conclusion that different strains of Lactobacillus and Bifidobacterium, in addition to a GFD, could restore the altered intestinal microbiota. Microbiota modulation therapy offers promising results in treating CD children; however, further research is required.

## Figures and Tables

**Table 1:** Microbiota alterations before CD onset in children with a genetic risk of CD.

Study	Subjects	Sample and Techniques	Microbiota Alterations
Pozo-Rubio et al. [24]	55 infants	Blood-sample-flow cytometry analysis Fecal sample quantitative PCR analysis	-Infants born through cesarean delivery: ↓ *B. catenulatum* ↑*B. angulatum* -Antibiotic use during pregnancy ↓*B. angulatum* -Formula feeding ↓*B. angulatum* -Antibiotic use during first the 4 months of life ↑*Bacteroides fragilis* ↑*B. angulatum* ↓*Bifidobacterium* spp. *B. longum* -Rotavirus vaccine ↓*Bacteroides fragilis* -Allergy and dermatitis ↓*B. angulatum*
Leonard et al. [25]	21 genetically predisposed infants 5 genetically non-predisposed infants	Fecal sample metagenomic analysis	-Standard and high risk of CD: ↓*Streptococcus* spp. ↓*Coprococcus* spp. ↓*Veillonella* spp. ↓*Parabacteroides* spp. ↓*Clostridium perfringens* ↑*Bacteroides* spp. ↑*Enterococcus* spp. -Infants born through cesarean delivery: ↓ *Bacteroides* spp. ↓*Parabacteroides* spp. ↑*Enterococcus faecalis* -Formula feeding ↓*Bifidobacterium breve* ↓*Staphylococcus epidermis* ↑*Bifidobacterium adolescentis* ↑*Ruminococcus gnavus* ↑*Lachnospiraceae bacterium* -Infant antibiotic exposure ↑*Bacteroides thetaiotaomicron* ↑*Propionibacterium* spp. ↑*Subdoligranulum* spp. ↓*Bifidobacterium merycicum* ↓*Streptococcus lutetiensis*
de Palma et al. [27]	20 infants	Fecal sample fluorescence in situ hybridization analysis	-High-risk infants ↑Gram-negative bacteria ↑*Bacteroides-Prevotella* group ↑*E. coli* ↑*Streptococcus-Lactococcus* spp. ↑*E. rectale-C. coccoides* -Sulfate-reducing bacteria ↑*C. lituseburense* ↑*C. histolyticum*
Olivares et al. [28]	22 infants	Fecal sample 16S rRNA gene pyrosequencing and real-time quantitative PCR analysis	-High-risk infants ↑ Bacillota phylum ↑Pseudomonadota phylum ↑*Corynebacterium* genus ↑*Gemella* genus ↑*Clostridium* sensu stricto ↑*Escherichia/Shigella* ↓Actinomycetota phylum ↓*Bifidobacterium* spp
Leonard et al. [23]	20 infants	Fecal sample analysis using shotgun sequencing and metabolomic profiling	↓*Bacteroides vulgatus* str_3775_S_1080 Branch ↓*Bacteroides uniformis*_ -American Type Culture Collection (ATCC)_8492 ↓*Streptococcus thermophiles* ↓*Faecalibacterium* *prausnitzii* ↓*Clostridium clostridioforme* ↓*Veillonella parvula* ↑*Dialister invisus* strain DSM_15470 ↑*Parabacteroides* species and strains ↑*Lachnospiraceae bacterium* ↑*Bifidobacterium longum* ↑*Bifidobacterium breve* ↑*Escherichia coli* ↑*Clostridium hathewayi* ↑*Eubacterium eligens*
Ghirdhar et al. [35]	33 children	Fecal sample 16S rRNA sequencing and flow cytometry analysis Blood plasma sample metabolomic analysis	↑IgA-coated bacteria and unique targets of IgA in their gut microbiota
Rintala et al. [43]	27 infants	Fecal sample 16S rRNA sequencing	No statistically significant differences in early microbiota composition between children that later developed CD and healthy controls were found
Olivares et al. [44]	127 infants	Fecal sample 16S rRNA sequencing	-High risk of CD, both formula and breastfeeding ↑ETEC -Formula feeding ↑*C. perfringens* ↑*C. difficile*

ETEC—enterotoxigenic *E. coli*; ↑ increased levels; ↓decreased levels.

**Table 2:** Gut microbiota alterations in children at CD onset.

Study	Subjects	Sample and Techniques	Microbiota Alterations	Other Findings
El Mouzan et al. [46]	20 CD children 20 fecal controls 19 mucosal controls		Duodenal samples of CD ↑Pseudomonadota phylum ↑*Lactobacillus acidophilus*, *Neisseria* spp. ↑*Coprococcus* spp. Fecal samples of CD ↑*Verucomicrobia* spp. ↑*Clostridium* spp. ↑*Escherichia* spp. ↑*Lachnospiraceae_bacterium_oral* ↓*Bifidobacterium* genus ↓*Bacteroides* spp.	Fecal samples were more diverse and richer in bacteria compared with mucosal samples Bacillota and Bacteroidota were the most abundant phyla in both fecal and mucosal samples
Zafeiropoulou et al. [47]	20 CD children 45 CD under GFD 57 healthy controls 19 children at risk of CD	Fecal sample 16S rRNA sequencing	Untreated CD ↓*Clostridium sensu stricto 1* genus ↓*Ruminococcus* genus	Microbial dysbiosis was not reported in CD compared to healthy controls *Alistipes* was correlated with the presence of symptoms of CD
Di Base et al. [50]	21 CD children 16 healthy controls	Fecal samples Duodenal sample 16S rRNA sequencing	Duodenal samples of CD ↑*Enterobacteriaceae* family ↑*Bacteroidetes/Streptococcus* spp. Fecal samples of CD ↓ *Bacteroides–Prevotella* ↓*Akkermansia* spp. ↓*Staphylococcaceae* family	Patients with abdominal pain ↑*Bacillaceae* family ↑*Enterobaeriaceae* family Patients with diarrhea ↓*Clostridium* cluster XIVa ↓*Akkermansia* ↑*Bacillaceae* ↑*Fusobacterium*
Schippa et al. [62]	20 CD children, before and after GFD 10 healthy controls	Duodenal sample 16S ribosomal DNA analysis compared with TTGE	In CD patients vs. controls ↑*Bacteroides vulgatus* ↑*Escherichia coli* Active CD vs. Inactive CD prevalence *B. vulgatus* (80% vs. 90%) *Clostridium coccoides* group (50% vs. 90%) *Bifidobacterium* spp (20% vs. 40%)	Mean interindividual similarity index: 54.9% ± 14.9% Active CD 55.6% ± 15.7% remission state 21.8% ± 30.16% controls Similarity index between CD children before and after GFD: 63.9% ± 15.8%
Sample et al. [63]	22 CD children, before and after GFD 17 healthy controls	Fecal sample16S ribosomal RNA sequencing	Active CD vs. Controls ↑*Haemophilus* genera ↑*Alistipes* genera ↑*Bacteroides* genera	
El Mouzan et al. [51]	40 CD children 39 controls	Fecal samples Duodenal sample metagenomic analysis of microbial DNA	Fecal samples of CD ↓*Bacteroides intestinalis* ↓*Burkholderiales bacterium 1-1-47* Mucosal samples of CD ↓*Human_endogenous_retrovirus_K*)	
El Mouzan et al. [61]	40 CD children 39 controls	Fecal samples Duodenal sample metagenomic analysis of microbial DNA	Fecal samples ↑ *Human polyomavirus 2, Enterobacteria phage mEpX1, Enterobacteria phage mEpX2*	Mucosal samples—no association with CD
El Mouzan et al. [55]	40 CD children 39 controls	Fecal samples Duodenal sample metagenomic analysis of microbial DNA	Fecal samples of CD ↓*Pichiaceae* family ↓*Pichia kudriavzevii* ↑*Saccharomycetes* family ↑*Saccharomyces cerevisiae* ↑*Tricholomataceae* family ↑Mucosal samples of CD ↑ *Saccharomycetaceae* family ↑*Candida* spp. ↓*Pneumocystis* spp. ↓*Pneumocystis jirovecii*	Fecal fungal communities were more abundant than those observed in mucosal samples
Sanchez et al. [64]	32 active CD on GFD 17 8 healthy controls	Duodenal mucosa sample 16S ribosomal RNA sequencing	Active CD ↑Pseudomonadota phylum ↑*Enterobacteriaceae* family ↑*Klebsiella oxytoca* ↑*Staphylococcus epidermidis* ↑*Staphylococcus pasteuri* ↓Bacillota phylum ↓*Streptococcaceae* family	Non-active CD ↑*Streptococcus mitis* group

TTGE—temporal temperature gradient gel electrophoresis; ↑ increased levels; ↓decreased levels.

**Table 3:** Gut microbiota alterations in CD patients under a GFD.

Study	Subjects	Sample and Techniques	Microbiota Alterations	Other Findings
Di Cagno [45]	19 CD children under GFD (T-CD) 15 non-celiac controls	Fecal samples Duodenal samples,-both on PCR and DGGE analysis	Duodenal biopsy ↑Eubacteria in T-CD Fecal samples of T-CD ↑*Bacteroides* spp. ↑*Staphylococcus* spp. ↑*Salmonella* spp. ↑*Shigella* spp. ↑*Klebsiella* spp.	
Sample et al. [63]	22 CD children before and after GFD 17 healthy controls	Fecal sample 16S ribosomal RNA sequencing	CD after GFD vs. controls ↑*Haemophilus* genera ↑*Alistipes* genera ↑*Bacteroides* genera ↑*Holdemania* genera ↑*Blautia* genera	*Faecalibacterium* and *Roseburia* were enriched in patients whose aTTG levels did not normalize after GFD

DGGE—denaturing gradient gel electrophoresis; ↑ increased levels; ↓decreased levels.

## Data Availability

Not applicable.

## References

[1] Withoff S., Li Y., Jonkers I., Wijmenga C. (2016). Understanding Celiac Disease by Genomics. Trends Genet..

[2] Prieto J., Singh K.B., Nnadozie M.C., Abdal M., Shrestha N., Abe R.A.M., Masroor A., Khorochkov A., Mohammed L. (2021). New Evidence in the Pathogenesis of Celiac Disease and Type 1 Diabetes Mellitus: A Systematic Review. Cureus.

[3] King J.A., Jeong J., Underwood F.E., Quan J., Panaccione N., Windsor J.W., Coward S., deBruyn J., Ronksley P.E., Shaheen A.-A. (2020). Incidence of Celiac Disease Is Increasing Over Time: A Systematic Review and Meta-analysis. Am. J. Gastroenterol..

[4] Roberts S.E., Morrison-Rees S., Thapar N., Benninga M.A., Borrelli O., Broekaert I., Dolinsek J., Martin-De-Carpi J., Mas E., Miele E. (2021). Systematic review and meta-analysis: The incidence and prevalence of paediatric coeliac disease across Europe. Aliment. Pharmacol. Ther..

[5] Van Berge-Henegouwen G.P., Mulder C.J. (1993). Pioneer in the gluten free diet: Willem-Karel Dicke 1905–1962, over 50 years of gluten free diet. Gut.

[6] Paulley J.W. (1954). Observation on the aetiology of idiopathic steatorrhoea; jejunal and lymphnode biopsies. BMJ.

[7] Berger E., Burgin-Wolff A., Freudenberg E. (1964). Diagnostisehe Bewertung des Nachweises yon Gliadin-AntikSrpern bei Cöliakie. Klin. Wochenschr..

[8] Falchuk Z.M., Rogentine G.N., Strober W. (1972). Predominance of histocompatibility antigen HLA8 in patients with gluten-sensitive enteropathy. J. Clin. Investig..

[9] Mearin M.L., Agardh D., Antunes H., Al-Toma A., Auricchio R., Castillejo G., Catassi C., Ciacci C., Discepolo V., Dolinsek J. (2022). ESPGHAN Position Paper on Management and pandemic. Acta Paediatr..

[10] Meeuwisse G.W. (1970). Diagnostic criteria in coeliac disease. Acta Paediatr. Scand..

[11] McNeish A.S., Harms H.K., Rey J., Shmerling D.H., Visakorpi J.K., A Walker-Smith J. (1979). The diagnosis of coeliac disease. A commentary on the current practices of members of the European Society for Paediatric Gastroenterology and Nutrition (ESPGAN). Arch. Dis. Child..

[12] Revised Criteria for Diagnosis of Coeliac Disease (1990). Report of Working Group of European Society of Paediatric Gastroenterology and Nutrition. Arch. Dis. Child..

[13] Bozomitu L., Miron I., Raileanu A.A., Lupu A., Paduraru G., Marcu F.M., Buga A.M.L., Rusu D.C., Dragan F., Lupu V.V. (2022). The Gut Microbiome and Its Implication in the Mucosal Digestive Disorders. Biomedicines.

[14] Lupu V.V., Miron I.C., Raileanu A.A., Starcea I.M., Lupu A., Tarca E., Mocanu A., Buga A.M.L., Lupu V., Fotea S. (2023). Difficulties in Adaptation of the Mother and Newborn via Cesarean Section versus Natural Birth-A Narrative Review. Life.

[15] Zugravu C., Nanu M.I., Moldovanu F., Arghir O.C., Mihai C.M., Oțelea M.R., Cambrea S.C. (2018). The influence of perinatal education on breastfeeding decision and duration. Int. J. Child Health Nutr..

[16] Wu X., Qian L., Liu K., Wu J., Shan Z. (2021). Gastrointestinal microbiome and gluten in celiac disease. Ann. Med..

[17] Tomer R., Patiyal S., Dhall A., Raghava G.P.S. (2023). Prediction of celiac disease associated epitopes and motifs in a protein. Front. Immunol..

[18] Liang C.P., Geng L.L., Chen P.Y., Li H.W., Ren L., Gong S.T. (2022). Celiac disease may be rare among children in South China. J. Int. Med. Res..

[19] Yuan J.L., Xu J., Shuai H., JinYan G., HongBing C. (2015). Recent advances in celiac disease. J. Food Saf. Food Qual..

[20] Valitutti F., Cucchiara S., Fasano A. (2019). Celiac Disease and the Microbiome. Nutrients.

[21] Lupu V.V., Adam Raileanu A., Mihai C.M., Morariu I.D., Lupu A., Starcea I.M., Frasinariu O.E., Mocanu A., Dragan F., Fotea S. (2023). The Implication of the Gut Microbiome in Heart Failure. Cells.

[22] Lin Z., Zu X.-P., Xie H.-S., Jin H.-Z., Yang N., Liu X.-R., Zhang W.-D. (2016). Research progress in mechanism of intestinal microorganisms in human diseases. Acta Pharm. Sin..

[23] Leonard M.M., Valitutti F., Karathia H., Pujolassos M., Kenyon V., Fanelli B., Troisi J., Subramanian P., Camhi S., Colucci A. (2021). Microbiome signatures of progression toward celiac disease onset in at-risk children in a longitudinal prospective cohort study. Proc. Natl. Acad. Sci. USA.

[24] Pozo-Rubio T., de Palma G., Mujico J.R., Olivares M., Marcos A., Acuña M.D., Polanco I., Sanz Y., Nova E. (2013). Influence of early environmental factors on lymphocyte subsets and gut microbiota in infants at risk of celiac disease; the PROFICEL study. Nutr. Hosp..

[25] Leonard M.M., Karathia H., Pujolassos M., Troisi J., Valitutti F., Subramanian P., Camhi S., Kenyon V., Colucci A., Serena G. (2020). Multi-omics analysis reveals the influence of genetic and environmental risk factors on developing gut microbiota in infants at risk of celiac disease. Microbiome.

[26] Hov J.R., Zhong H., Qin B., Anmarkrud J.A., Holm K., Franke A., Lie B.A., Karlsen T.H. (2015). The influence of the autoimmunity-associated ancestral HLA haplotype AH8. 1 on the human gut microbiota: A cross-sectional study. PLoS ONE.

[27] De Palma G., Capilla A., Nadal I., Nova E., Pozo T., Varea V., Polanco I., Castillejo G., López A., Garrote J. (2010). Interplay between human leukocyte antigen genes and the microbial colonization process of the newborn intestine. Curr. Issues Mol. Biol..

[28] Olivares M., Neef A., Castillejo G., De Palma G., Varea V., Capilla A., Palau F., Nova E., Marcos A., Polanco I. (2014). The HLADQ2 genotype selects for early intestinal microbiota composition in infants at high risk of developing coeliac disease. Gut.

[29] Yoshida N., Emoto T., Yamashita T., Watanabe H., Hayashi T., Tabata T., Hoshi N., Hatano N., Ozawa G., Sasaki N. (2018). Bacteroides vulgatus and Bacteroides dorei reduce gut microbial lipopolysaccharide production and inhibit atherosclerosis. Circulation.

[30] Bayer A.L., Fraker C.A. (2017). The folate cycle as a cause of natural killer cell dysfunction and viral etiology in type 1 diabetes. Front. Endocrinol..

[31] Chua H.H., Chou H.C., Tung Y.L., Chiang B.L., Liao C.C., Liu H.H., Ni Y.H. (2018). Intestinal dysbiosis featuring abundance of Ruminococcus gnavus associates with allergic diseases in infants. Gastroenterology.

[32] Olshan K.L., Leonard M.M., Serena G., Zomorrodi A.R., Fasano A. (2020). Gut microbiota in Celiac Disease: Microbes, metabolites, pathways and therapeutics. Expert Rev. Clin. Immunol..

[33] Zeng H., Ishaq S.L., Zhao F.-Q., Wright A.-D.G. (2016). Colonic inflammation accompanies an increase of β-catenin signaling and *Lachnospiraceae*/*Streptococcaceae bacteria* in the hind gut of high-fat diet-fed mice. J. Nutr. Biochem..

[34] Cabral D., Penumutchu S., Reinhart E.M., Zhang C., Korry B.J., Wurster J.I., Nilson R., Guang A., Sano W.H., Rowan-Nash A.D. (2019). Microbial metabolism modulates antibiotic susceptibility within the murine gut microbiome. Cell Metab..

[35] Girdhar K., Dogru Y.D., Huang Q., Yang Y., Tolstikov V., Raisingani A., Chrudinova M., Oh J., Kelley K., Ludvigsson J. (2023). Dynamics of the gut microbiome, IgA response, and plasma metabolome in the development of pediatric celiac disease. Microbiome.

[36] Maffeis C., Martina A., Corradi M., Quarella S., Nori N., Torriani S., Plebani M., Contreas G., Felis G.E. (2016). Association between intestinal permeability and faecal microbiota composition in Italian children with beta cell autoimmunity at risk for type 1 diabetes. Diabetes Metab. Res. Rev..

[37] Stewart C.J., Ajami N.J., O’brien J.L., Hutchinson D.S., Smith D.P., Wong M.C., Ross M.C., Lloyd R.E., Doddapaneni H., Metcalf G.A. (2018). Temporal development of the gut microbiome in early childhood from the TEDDY study. Nature.

[38] Ye Z., Zhang N., Wu C., Zhang X., Wang Q., Huang X., Du L., Cao Q., Tang J., Zhou C. (2018). A metagenomic study of the gut microbiome in Behcet’s disease. Microbiome.

[39] Kameyama K., Itoh K. (2014). Intestinal colonization by a Lachnospiraceae bacterium contributes to the development of diabetes in obese mice. Microbes Environ..

[40] Nakanishi Y., Sato T., Ohteki T. (2015). Commensal Gram-positive bacteria initiates colitis by inducing monocyte/macrophage mobilization. Mucosal Immunol..

[41] Deleu S., Machiels K., Raes J., Verbeke K., Vermeire S. (2021). Short chain fatty acids and its producing organisms: An overlooked therapy for IBD?. eBioMedicine.

[42] Lauwers G.Y., Fasano A., Brown I.S. (2015). Duodenal lymphocytosis with no or minimal enteropathy: Much ado about nothing?. Mod. Pathol..

[43] Rintala A., Riikonen I., Toivonen A., Pietilä S., Munukka E., Pursiheimo J.-P., Elo L.L., Arikoski P., Luopajärvi K., Schwab U. (2018). Early fecal microbiota composition in children who later develop celiac disease and associated autoimmunity. Scand. J. Gastroenterol..

[44] Olivares M., Benítez-Páez A., De Palma G., Capilla A., Nova E., Castillejo G., Varea V., Marcos A., Garrote J.A., Polanco I. (2018). Increased prevalence of pathogenic bacteria in the gut microbiota of infants at risk of developing celiac disease: The PROFICEL study. Gut Microbes.

[45] Di Cagno R., De Angelis M., De Pasquale I., Ndagijimana M., Vernocchi P., Ricciuti P., Gagliardi F., Laghi L., Crecchio C., Guerzoni M.E. (2011). Duodenal and faecal microbiota of celiac children: Molecular, phenotype and metabolome characterization. BMC Microbiol..

[46] El Mouzan M., Al-Hussaini A., Serena G., Assiri A., Al Sarkhy A., Al Mofarreh M., Alasmi M., Fasano A. (2022). Microbiota profile of new-onset celiac disease in children in Saudi Arabia. Gut Pathog..

[47] Zafeiropoulou K., Nichols B., Mackinder M., Biskou O., Rizou E., Karanikolou A., Clark C., Buchanan E., Cardigan T., Duncan H. (2020). Alterations in Intestinal Microbiota of Children with Celiac Disease at the Time of Diagnosis and on a Gluten-free Diet. Gastroenterology.

[48] Han T., Li J. (2021). Gut microbiota as a new player in children with celiac disease. J. Gastroenterol. Hepatol..

[49] Wacklin P., Kaukinen K., Tuovinen E., Collin P., Lindfors K., Partanen J., Mäki M., Mättö J. (2013). The duodenal microbiota composition of adult celiac disease patients is associated with the clinical manifestation of the disease. Inflamm. Bowel Dis..

[50] Di Biase A.R., Marasco G., Ravaioli F., Dajti E., Colecchia L., Righi B., D’Amico V., Festi D., Iughetti L., Colecchia A. (2020). Gut microbiota signatures and clinical manifestations in celiac disease children at onset: A pilot study. J. Gastroenterol. Hepatol..

[51] El Mouzan M., Assiri A., Al Sarkhy A. (2023). Gut microbiota predicts the diagnosis of celiac disease in Saudi children. World J. Gastroenterol..

[52] Grandi N., Tramontano E. (2018). Human Endogenous Retroviruses Are Ancient Acquired Elements Still Shaping Innate Immune Responses. Front. Immunol..

[53] El Mouzan M.I., Korolev K.S., Al Mofarreh M.A., Menon R., Winter H.S., Al Sarkhy A.A., Dowd S.E., Al Barrag A.M., Assiri A.A. (2018). Fungal dysbiosis predicts the diagnosis of pediatric Crohn’s disease. World J. Gastroenterol..

[54] Santelmann H., Howard J.M. (2005). Yeast metabolic products, yeast antigens and yeasts as possible triggers for irritable bowel syndrome. Eur. J. Gastroenterol. Hepatol..

[55] El Mouzan M., Al-Hussaini A., Fanelli B., Assiri A., AlSaleem B., Al Mofarreh M., Al Sarkhy A., Alasmi M. (2021). Fungal Dysbiosis in Children with Celiac Disease. Dig. Dis. Sci..

[56] Granito A., Zauli D., Muratori P., Grassi A., Bortolotti R., Petrolini N., Veronesi L., Gionchetti P., Bianchi F.B., Volta U. (2005). Anti-Saccharomyces cerevisiae and perinuclear anti-neutrophil cytoplasmic antibodies in coeliac disease before and after gluten-free diet. Aliment. Pharmacol. Ther..

[57] Mallant-Hent R.C., Mary B., Von Blomberg E., Yüksel Z., Wahab P.J., Gundy C., Meyer G.A., Mulder C.J.J. (2006). Disappearance of anti-saccharomyces cerevisiae antibodies in coeliac disease during a gluten-free diet. Eur. J. Gastroenterol. Hepatol..

[58] Nieuwenhuizen W.F., Pieters R.H., Knippels L.M., Jansen M.C., Koppelman S.J. (2003). Is Candida albicans a trigger in the onset of coeliac disease?. Lancet.

[59] Corouge M., Loridant S., Fradin C., Salleron J., Damiens S., Moragues M.D., Souplet V., Jouault T., Robert R., Dubucquoi S. (2015). Humoral immunity links Candida albicans infection and celiac disease. PLoS ONE.

[60] Lerner A., Matthias T. (2020). Candida albicans in celiac disease: A wolf in sheep’s clothing. Autoimmune Rev..

[61] El Mouzan M., Assiri A., Al Sarkhy A., Alasmi M., Saeed A., Al-Hussaini A., AlSaleem B., Al Mofarreh M. (2022). Viral dysbiosis in children with new-onset celiac disease. PLoS ONE.

[62] Schippa S., Iebba V., Barbato M., Di Nardo G., Totino V., Checchi M.P., Longhi C., Maiella G., Cucchiara S., Conte M.P. (2010). A distinctive ‘microbial signature’ in celiac pediatric patients. BMC Microbiol..

[63] Sample D.M., Fouhse J., King S.M., Huynh H.Q.M., Dieleman L.A.M., Willing B.P., Turner J.M. (2021). Baseline Fecal Microbiota in Pediatric Patients with Celiac Disease Is Similar to Controls but Dissimilar After 1 Year on the Gluten-Free Diet. JPGN Rep..

[64] Sánchez E., Donat E., Ribes-Koninckx C., Fernández-Murga M.L., Sanz Y. (2013). Duodenal-mucosal bacteria associated with celiac disease in children. Appl. Environ. Microbiol..

[65] Logan K., Perkin M.R., Marrs T., Radulovic S., Craven J., Flohr C., Bahnson H.T., Lack G. (2020). Early gluten introduction and celiac disease in the EAT study: A prespecified analysis of the EAT randomized clinical trial. JAMA Pediatr..

[66] Hummel S., Pflüger M., Hummel M., Bonifacio E., Ziegler A.G. (2011). Primary dietary intervention study to reduce the risk of islet autoimmunity in children at increased risk for type 1 diabetes: The BABYDIET study. Diabetes Care.

[67] Beyerlein A., Chmiel R., Hummel S., Winkler C., Bonifacio E., Ziegler A.G. (2014). Timing of gluten introduction and islet autoimmunity in young children: Updated results from the BABYDIET study. Diabetes Care.

[68] Sellitto M., Bai G., Serena G., Fricke W.F., Sturgeon C., Gajer P., White J.R., Koenig S.S.K., Sakamoto J., Boothe D. (2012). Proof of concept of microbiome-metabolome analysis and delayed gluten exposure on celiac disease autoimmunity in genetically at-risk infants. PLoS ONE.

[69] Lionetti E., Castellaneta S., Francavilla R., Pulvirenti A., Tonutti E., Amarri S., Barbato M., Barbera C., Barera G., Bellantoni A. (2014). Introduction of gluten, HLA status, and the risk of celiac disease in children. N. Engl. J. Med..

[70] Vriezinga S.L., Auricchio R., Bravi E., Castillejo G., Chmielewska A., Escobar P.C., Kolaček S., Koletzko S., Korponay-Szabo I.R., Mummert E. (2014). Randomized feeding intervention in infants at high risk for celiac disease. N. Engl. J. Med..

[71] Aronsson C.A., Lee H.-S., Hårdaf Segerstad E.M., Uusitalo U., Yang J., Koletzko S., Liu E., Kurppa K., Bingley P.J., Toppari J. (2019). Association of gluten intake during the first 5 years of life with incidence of celiac disease autoimmunity and celiac disease among children at increased risk. JAMA.

[72] Mårild K., Dong F., Lund-Blix N.A., Seifert J., Barón A.E., Waugh K.C., Taki I., Størdal K., Tapia G., Stene L.C. (2019). Gluten intake and risk of celiac disease: Long-term follow-up of an At-risk birth cohort. Am. J. Gastroenterol..

[73] Lund-Blix N.A., Marild K., Tapia G., Norris J.M., Stene L.C., Stordal K. (2019). Gluten intake in early childhood and risk of celiac disease in childhood: A nationwide cohort study. Am. J. Gastroenterol..

[74] Aronsson C.A., Agardh D. (2023). Intervention strategies in early childhood to prevent celiac disease-a mini-review. Front. Immunol..

[75] Hakansson A., Andren Aronsson C., Brundin C., Oscarsson E., Molin G., Agardh D. (2019). Effects of lactobacillus plantarum and lactobacillus paracasei on the peripheral immune response in children with celiac disease autoimmunity: A randomized, double blind, placebo-controlled clinical trial. Nutrients.

[76] Ahrén I.L., Berggren A., Teixeira C., Martinsson Niskanen T., Larsson N. (2020). Evaluation of the efficacy of Lactobacillus plantarum HEAL9 and Lactobacillus paracasei 8700:2 on aspects of common cold infections in children attending daycare: A randomised, double-blind, placebo-controlled clinical study. Eur. J. Nutr..

[77] Lindfors K., Lin J., Lee H.-S., Hyoty H., Nykter M., Kurppa K., Liu E., Koletzko S., Rewers M., Hagopian W. (2019). Metagenomics of the faecal virome indicate a cumulative effect of enterovirus and gluten amount on the risk of coeliac disease autoimmunity in genetically at risk children: The TEDDY study. Gut.

[78] Stene L.C., Honeyman M.C., Hoffenberg E., Haas J.E., Sokol R.J., Emery L., Taki I., Norris J.M., Erlich H.A., Eisenbarth G.S. (2006). Rotavirus infection frequency and risk of celiac disease autoimmunity in early childhood: A longitudinal study. Am. J. Gastroenterol..

[79] Kemppainen K.M., Lynch K.F., Liu E., Lönnrot M., Simell V., Briese T., Koletzko S., Hagopian W., Rewers M., She J.X. (2017). Factors that increase risk of celiac disease autoimmunity after a gastrointestinal infection in early life. Clin. Gastroenterol. Hepatol..

[80] Quagliariello A., Aloisio I., Cionci N.B., Luiselli D., D’Auria G., Martinez-Priego L., Pérez-Villarroya D., Langerholc T., Primec M., Mičetić-Turk D. (2016). Effect of Bifidobacterium breve on the Intestinal Microbiota of Coeliac Children on a Gluten Free Diet: A Pilot Study. Nutrients.

[81] Klemenak M., Dolinšek J., Langerholc T., Di Gioia D., Mičetić-Turk D. (2015). Administration of Bifidobacterium breve Decreases the Production of TNF-α in Children with Celiac Disease. Dig. Dis. Sci..

[82] Olivares M., Castillejo G., Varea V., Sanz Y. (2014). Double-blind, randomised, placebo-controlled intervention trial to evaluate the effects of Bifidobacterium longum CECT 7347 in children with newly diagnosed coeliac disease. Br. J. Nutr..

[83] Primec M., Klemenak M., Di Gioia D., Aloisio I., Cionci N.B., Quagliariello A., Gorenjak M., Mičetić-Turk D., Langerholc T. (2018). Clinical intervention using Bifidobacterium strains in celiac disease children reveals novel microbial modulators of TNF-α and short-chain fatty acids. Clin. Nutr..

[84] Drabińska N., Jarocka-Cyrta E., Markiewicz L.H., Krupa-Kozak U. (2018). The Effect of Oligofructose-Enriched Inulin on Faecal Bacterial Counts and Microbiota-Associated Characteristics in Celiac Disease Children Following a Gluten-Free Diet: Results of a Randomized, Placebo-Controlled Trial. Nutrients.

